# Endocannabinoid levels in female-sexed individuals with diagnosed depression: a systematic review

**DOI:** 10.1186/s12905-024-03168-y

**Published:** 2024-06-17

**Authors:** Meagan McWhirter, Andrea Bugarcic, Amie Steel, Janet Schloss

**Affiliations:** 1 National Centre for Naturopathic Medicine, Faculty of Health, Southern Cross University, Lismore, NSW 2480 Australia; 2 ACCRIM, The University of Technology Sydney, Ultimo, NSW Australia

**Keywords:** Major depressive disorder, Depression, Endocannabinoid, Endocannabinoid system, Female, Systematic review

## Abstract

**Background:**

Major depressive disorder (MDD) is a highly prevalent mental health disorder with females experiencing higher rates of depression (11.6%), anxiety (15.7%) and physiological distress (14.5%) than males. Recently, the Endocannabinoid system (ECS) has been proposed to be a key contributing factor in the pathogenesis and symptom severity of MDD due to its role in neurotransmitter production, inflammatory response and even regulation of the female reproductive cycle. This review critically evaluates evidence regarding ECS levels in female-sexed individuals with depressive disorders to further understand ECS role.

**Materials and methods:**

A systematic literature review of available research published prior to April 2022 was identified using PubMed (U.S. National Library of Medicine), CINAHL (EBSCO), Web of Science, AMED and Scopus (Elsevier). Studies were included if they reported ECS analysis of female-sexed individuals with depression and were excluded if they did not differentiate results between sexes, assessed mental health conditions other than depression, tested efficacy of endocannabinoid/n-acylethanolamine/cannabis or marijuana administration and that were unable to be translated. Critical appraisal of each included study was undertaken using the Joanna Briggs Institute Critical Appraisal Tool for Systematic Reviews.

**Results:**

The 894 located citations were screened for duplicates (*n* = 357) and eligibility by title and abstract (*n* = 501). The full text of 33 studies were reviewed, and 7 studies were determined eligible for inclusion. These studies indicated that depressed female-sexed individuals have altered levels of ECS however no significant pattern was identified due to variability of study outcomes and measures, limiting overall interpretation.

**Discussion:**

This review suggests potential involvement of ECS in underlying mechanisms of MDD in female sexed-individuals, however no pattern was able to be determined. A major contributor to the inability to attain reliable and valid understanding of the ECS levels in female-sexed individuals with depression was the inconsistency of depression screening tools, inclusion criteria’s and analysis methods used to measure eCBs. Future studies need to implement more standardised methodology to gain a deeper understanding of ECS in female-sexed individuals with depressive disorders.

**Trial registration:**

This review was submitted to PROSPERO for approval in April 2022 (Registration #CRD42022324212).

## Introduction

Major depressive disorder (MDD) is a highly prevalent mental health disorder, characterised by both cognitive and physiological symptoms including depressed mood, loss of interest and pleasure, sleep disturbance, recurrent thoughts of death and sense of worthlessness [1]. Between 2017 and 2018, 10.4% of the Australian population was affected by depression, with females experiencing higher rates of depression (11.6%), anxiety (15.7%) and physiological distress (14.5%) than males [2]. The DSM-V criteria is typically used by mental health professionals to diagnose MDD, with the patient required to meet five or more of the criteria, including depressed mood, loss of interest and pleasure, sleep disturbance, recurrent thoughts of death and sense of worthlessness [3], to be diagnosed with MDD. Females diagnosed with MDD typically experience longer symptom presentation, increased recurrence of depressive episodes, have a lower quality of life and experience a wider range of side effects from typical anti-depressant medication such as weight gain, anxiety [4] and sexual dysfunction [5]. Some studies have indicated SSRIs and tricyclic- antidepressants are less well-tolerated in females and they respond more slowly to this treatment than males [6]. It has been suggested that such sex-based differences in presentation of depressive symptoms and treatment response is due to genetic, biological and environmental factors [6].

While the pathogenesis of MDD is still poorly understood, there is agreement that influences vary from chronic stress to biochemical abnormalities, increased inflammation and the female reproductive cycle [6–8]. More recently, impaired functioning of the endocannabinoid system (ECS) has been suggested to be a key contributing factor in the pathogenesis and symptom severity of MDD due to the ECS’ role in neurotransmitter production, inflammatory response and even regulation of the female menstrual cycle [9]. The ECS is composed of endogenous endocannabinoids (eCBs) (anandamide (AEA), 2-arachidonoyl-glycerol (2-AG) [10] and N-acylethanolamines (NAEs) which are fatty acid amides and include Palmitoylethanolamide (PEA), Oleoylethanolamide (OEA) and Stearolyethanolamide (SEA) [11]. There is also a variety of receptors expressed within the ECS including CB1 and CB2 receptors. Interaction between eCBs, NAEs and receptors, expressed in the central nervous system (CNS), alter release of neurotransmitters at GABAergic synapses and modulate acetylcholine and serotonin (5-HT) pathways implicated in mood disorders, such as MDD [12]. However, current research investigating the involvement of CB1 receptors in depression and mood disorders is conflicting. For example, post-mortem findings of individuals with MDD and those who have died by suicide have shown an increased concentration and signalling of CB1 receptors in the prefrontal cortex [13, 14], while decreased CB1 receptor density was found in MDD patients treated with SSRIs compared to healthy controls, suggesting that pharmaceutical treatment may alter expression of CB1 receptors [15].

Further, studies measuring two most prominent endocannabinoids, AEA and 2-AG, reported lower levels of these eCBs in depressive patients [10, 16], possibly to suppress their down regulation of inflammatory cytokines such as TNF-α and IL-6 [17] linked to increased depressive symptoms [18, 19]. PEA and OEA are fatty acid amides which may regulate mood and neurosteroid biosynthesis of allopregnanolone [11, 20] a potential hormone involved in the pathogenesis of postnatal depression (PND), with lower levels suggested to influence symptom severity [17, 21]. Finally, SEA may enhance synaptic vesicle release at GABAergic and glutamatergic synapses leading to anti-inflammatory and neurological protective effects [22]. Because of these diverse actions, the ECS is being investigated as a possible therapeutic target for MDD. Pre-clinical reports suggest that modulation of the ECS displays antidepressant and anxiolytic effects similar to pharmaceutical anti-depressants by enhancing serotonergic and noradrenergic transmission [23]. Further, possible modulation of the ECS with cannabidiol (CBD), which targets the CB1 receptors within the ECS, may lead to anti-depressant and anxiolytic properties [24].

While research into ECS shows promise as a therapeutic target for mood disorders in the general population, there is limited research exploring the role of eCBs in female-sexed individuals with depression. Given the well documented differences between male and female sexed individuals response to pharmacologic treatment of MDD, further exploration into underlying mechanisms of MDD in female-sexed individuals is warranted. This systematic review seeks to understand eCBs and NAE levels in female-sexed individuals with diagnosed depression, to further our understanding of the potential of the ECS as a therapeutic target for this condition. A note on language-we understand that sex and gender can be complex, therefore the term *female* in this review describes female-sexed individuals and does not refer to gender.

## Materials and methods

### Main outcome

To examine the levels of endocannabinoids and n-acylethanolamines in female-sexed individuals diagnosed with depression.

### Additional outcome

To assess if different levels of endocannabinoids and n-acylethanolamines are present in female-sexed individuals with depression compared to healthy female-sexed individuals.

A systematic review protocol was developed and submitted to PROSPERO for approval in April 2022 (Registration #CRD42022324212). All authors contributed to the development of search terms and eligibility criteria. A medical librarian was consulted to assisted with the development of search terms and database searching. Search terms were divided into four groups and then combined within the search. Refer to Table 1.

**Table 1:** Search Strategy used for PUBMED (U.S National Library of Medicine)

Group 1	Women [Mesh] OR female*
Group 2	Endocannabinoid* OR “Endogenous Cannabinoid*” OR eCB OR N-acylethanolamine OR anadamide OR 2-arachidonoyl OR palmitoylethanolamide OR oleoylethanolamide OR stearoylethanolamide
Group 3	Depress* OR MDD
Group 4	Cannabis OR Marijuana
Final Search	#1 AND #2 AND #3 NOT #4

### Databases

A literature search was conducted on the following databases: PubMed U.S. National Library of Medicine, CINAHL (EBSCO), Web of Science, AMED and Scopus (Elsevier) (Tables 1, 2, 3, 4 and 5).

**Table 2:** Search Strategy used for CINAHL (EBSCO)

Group 1	Wom? *n* OR female*
Group 2	Endocannabinoid* OR “Endogenous Cannabinoid*” OR eCB OR N-acylethanolamine OR anadamide OR 2-arachidonoyl OR palmitoylethanolamide OR oleoylethanolamide OR stearoylethanolamide
Group 3	Depress* OR MDD
Group 4	Cannabis OR Marijuana
Final Search	S1 AND S2 AND S3 NOT S4

**Table 3:** Search Strategy used for Web of Science

Group 1	Wom? *n* OR female*
Group 2	Endocannabinoid* OR “Endogenous Cannabinoid*” OR eCB OR N-acylethanolamine OR anadamide OR 2-arachidonoyl OR palmitoylethanolamide OR oleoylethanolamide OR stearoylethanolamide
Group 3	Depress* OR MDD
Group 4	Cannabis OR Marijuana
Final Search	#1 AND #2 AND #3 NOT #4

**Table 4:** Search Strategy used for AMED

Group 1	Wom#*n* OR female*
Group 2	Endocannabinoid* OR “Endogenous Cannabinoid*” OR eCB OR N-acylethanolamine OR anadamide OR 2-arachidonoyl OR palmitoylethanolamide OR oleoylethanolamide OR stearoylethanolamide
Group 3	Depress* OR MDD
Group 4	Cannabis OR Marijuana
Final Search	S1 AND S2 AND S3 NOT S4

**Table 5:** Search Strategy used for SCOPUS (Elsevier)

Group 1	Wom? *n* OR female*
Group 2	Endocannabinoid* OR “Endogenous Cannabinoid*” OR eCB OR N-acylethanolamine OR anadamide OR 2-arachidonoyl OR palmitoylethanolamide OR oleoylethanolamide OR stearoylethanolamide
Group 3	Depress* OR MDD
Group 4	Cannabis OR Marijuana
Final Search	#1 AND #2 AND #3 AND NOT #4

### Eligibility criteria

This review included original research published up to April 2022, which examined eCB levels in female-sexed individuals. An updated literature search was conducted in March 2023, however no new articles which met the eligibility criteria were available. Articles were eligible for inclusion if they reported the results of original research studies measuring blood or hair levels of endocannabinoids and n-acylethanolamines in female-sexed individuals aged 18 years or older with depression (inclusive of mild, moderate, major depression, antenatal depression, postnatal depression and post-traumatic stress disorder). PTSD was deemed a suitable addition to inclusion criteria as depression is also often present, further, females with PTSD and comorbid depression are at a greater risk of death to those with no depression or trauma exposure. Depression among study participants must have been confirmed via diagnosis by a health professional or through application of a validated mental-health instrument.

Articles were excluded from this review if they reported the results of studies that did not include differentiation of results between sexes or included individuals born male-sexed who had transitioned to female-sexed later in life. Results from studies sampling populations with severe psychiatric conditions including schizophrenia, obsessive compulsion disorder and bipolar disorder, studies testing efficacy of endocannabinoid/n-acylethanolamine/cannabis or marijuana administration were also excluded. Studies in a language other than English that were unable to be translated by the research team and their networks or by Google Translate were also deemed ineligible.

## Results

### Study selection and data extraction

**Fig. 1:**
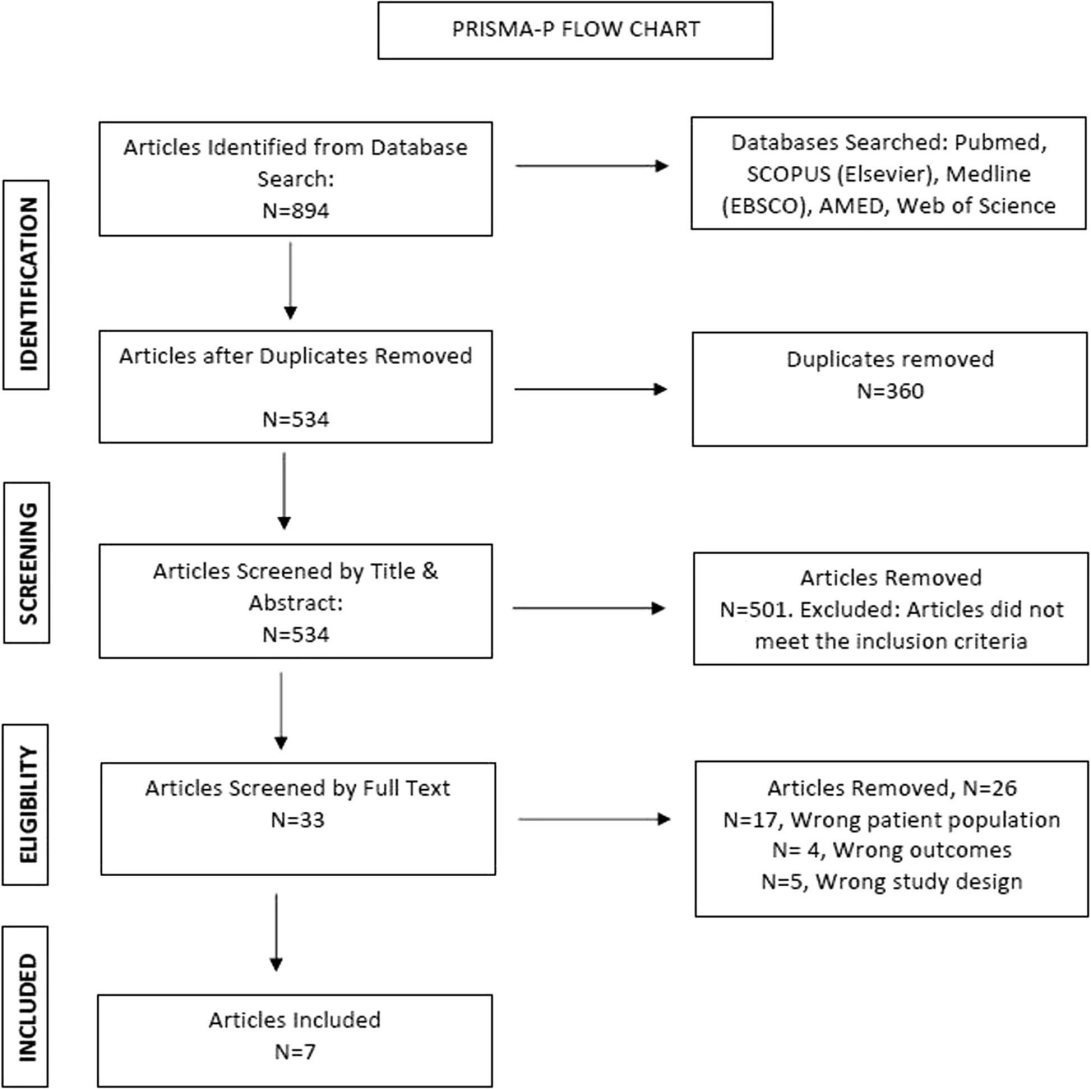
PRISMA-P flowchart of study identification and selection process

The initial literature search identified 894 studies. After removal of 360 duplicates, each study was screened by title and abstract, in which a further 501 were removed. The remaining studies underwent full text screening by two reviewers (M.M, J.S) which resulted in seven articles that met the full inclusion criteria of this review. The article selection process is outlined in Fig. 1.

### Assessment of risk of bias and data summary table

Each eligible article was critically appraised for methodological quality and risk of bias using the Joanna Briggs Institute Critical Appraisal Tool for Systematic Reviews [25]. The checklist for case-control studies was used to assess all seven studies (Table 6). Overall, the appraisals found reliable methodology in all studies and none were excluded from this review. However, limitations were present in some studies. Barry et al. [26] did not identify confounding factors, while Gao et al. [27] compared eCB levels between groups with varying levels of depression rather than against a healthy control group.

**Table 6:** Critical appraisal table (Case- control)

The Joanna Briggs Institute Critical Appraisal Tool for Systematic Reviews: Checklist for Case Control Studies										
Authors/date	(1)	(2)	(3)	(4)	(5)	(6)	(7)	(8)	(9)	(10)
Barry [26]	Y	Y	Y	Y	Y	N	N	Y	Y	Y
Gao [27]	Y	N	N	Y	Y	Y	Y	Y	Y	Y
Hill [28]	Y	Y	Y	Y	Y	Y	Y	Y	Y	Y
Hill [29]	Y	Y	Y	Y	Y	Y	Y	Y	Y	Y
Ho [30]	Y	Y	Y	Y	Y	Y	Y	Y	Y	Y
Stensson [32]	Y	Y	Y	Y	Y	Y	Y	Y	Y	Y
Stensson [31]	Y	Y	Y	Y	Y	Y	Y	Y	Y	Y

(1) Were the groups comparable other than the presence of disease in cases or the absence of disease in controls?

(2) Were cases and controls matched appropriately?

(3) Were the same criteria used for identification of cases and controls?

(4) Was exposure measured in a standard, valid and reliable way?

(5) Was exposure measured in the same way for cases and controls?

(6) Were confounding factors identified?

(7) Were strategies to deal with confounding factors stated?

(8) Were outcomes assessed in a standard, valid and reliable way for cases and controls?

(9) Was the exposure period of interest long enough to be meaningful?

(10) Was appropriate statistical analysis used?

Key: *Y* yes, *N* no, *UC* Unclear, *N/A* Not applicable

### Study characteristics

All studies assessed female individuals only, except Gao et al. [27] which had a study cohort of *n* = 38 male and *n* = 169 female individuals and used sex-stratified analysis of results to determine eCBs in female individuals. Matched control groups with no lifetime history of mood disorders were included in Hill et al. [28] (*n* = 55), Hill et al. [29] (*n* = 30) and Ho et al. [30] (*n* = 55). Barry et al. [26] (*n* = 17) compared their cases to those with no presence of burning mouth syndrome, while studies conducted by Stensson et al. in 2018 [32] (*n* = 220) and 2020 [31] (*n* = 70) included females with fibromyalgia. Gao et al. [27] compared eCB levels between groups of low and high depression rather than a healthy population, while Hill et al. [28] compared females with either minor or major depression. The number of participants across studies ranged from *n* = 17 to *n* = 220. None of the identified studies included participants with comorbid substance use including cannabis, alcohol or drugs such as cocaine and amphetamines. An overview of these studies characteristics can be viewed in Table 7.

**Table 7:** Summary of study characteristics and results assessing eCB levels in female-sexed individuals with depression and mood disorders

First Author	Year	Study Design	Participants	Outcome Measure	Endocannabinoid Analysis Method	Inclusion/ Exclusion Criteria Study Group	Inclusion/ Exclusion Criteria Comparison Group
Barry [26]	2018	Case control study	17 females aged 36–75 Condition: BMS & depression	QIDS-SR16	LC-MS/MS	***Inclusion***: primary complaint of oral cavity burning pain, with an absence of clinical signs determined by 2 independent dentists ***Exclusions***: systemic or oral disease, tongue injury, contact stomatis, vitamin deficiency, diabetes & connective tissue disorders	***Inclusion***: absence of clinical signs of oral cavity pain
Gao [27]	2020	Case control study	207 participants, 38 male and 169 females aged 41.81 ± 11.41	German version of the PHQ-9 GAD-7	LC-MS/MS	***Inclusion***: Aged 18–68 years, adequate language skills (capable of reading/writing in German), PHQ-9 total score > 15 (moderate to severe major depression)	***Inclusion***: PHQ-9 total score < 15 (no or mild depression)
Hill [28]	2008	Case control study	55 females aged over 18 years Condition: Major and minor depression	DSM-IV criteria for clinical depression & interview by trained persons using DISH	LC-APCI-MS	***Inclusion***: met the DSM-IV criteria for either current major or minor depressive episode, no hx of chronic medical conditions, no acute infectious disease, no prescribed medication in the past 6 months including anti-depressants ***Exclusion***: hx of/or current diagnosis of co-morbid psychotic, eating disorder, alcohol, substance abuse or anxiety disorder, > 55 years, pregnant in the past year, were menopausal/postmenopausal/irregular menses, undernourished (serum albumin < 3.3 g/dL) or use of illicit substances including cannabis, cocaine and heroin	***Inclusion***: match study participant in terms of age & ethnicity, lifetime free hx of medical and psychiatric illness and score < 5 on 10-item centre for Epidemiologic studies depression scale
Hill [29]	2009	Case control study	30 females aged over 18 years Condition: Major depression	DSM-IV criteria for current major depressive episode & interview by trained persons using DISH	LC-APCI-MS	***Inclusion***: met the DSM-IV criteria for current major depressive episode, no prescribed medication in the past 6 months including anti-depressants ***Exclusion***: hx of/or current diagnosis of co-morbid psychotic, eating disorder, alcohol, substance abuse or anxiety disorder	***Inclusion***: match study participant in terms of age & ethnicity, lifetime free hx of medical and psychiatric illness and score < 5 on 10-item centre for Epidemiologic studies depression scale
Ho [30]	2012	Case control study	55 females aged over 18 Condition: Depression	Validated physician diagnosis by clinical interview for DSM-IV	LC-APCI-MS	***Inclusion***: met the DSM-IV criteria for current major depressive episode, no prescribed medication in the past 6 months including anti-depressants ***Exclusion***: Older then 55, menopausal/postmenopausal, pregnant in the last year, irregular menses, undernourished (serum albumin ≤3.3 g/dL, use of cannabis/cocaine/heroin	***Inclusion***: match study participant in terms of age & ethnicity, lifetime free hx of medical and psychiatric illness
Stensson [32]	2018	Case control study	220 females aged 20–65 Condition: FM and depression	HADS	LC-MS/MS	***Inclusion***: diagnosis of FM under ACR-1990 ***Exclusion***: high BP (> 160/90 mmHg), osteoarthritis in knee/hip, severe somatic/psychiatric disorder, excess consumption of alcohol, regular resistance or relaxation therapy, inability to not use analgesics/NSAIDS/hypnotics 48 h prior to examination	***Inclusion***: no current pain
Stensson[31]	2020	Non-randomised controlled study	70 females aged between 20–65 Condition: FM & depression	HADS	LC-MS/MS	***Inclusion***: diagnosis of FM under ACR-1990 ***Exclusion***: high BP (> 160/90 mmHg), osteoarthritis in knee/hip, severe somatic/psychiatric disorder, excess consumption of alcohol, regular resistance or relaxation therapy, inability to not use analgesics/NSAIDS/hypnotics 48 h prior to examination	***Inclusion***: no current pain

KEY: *BDI *Beck Depression inventory, *BDI-2 *Beck depression inventory- 2nd Edition, *BP *Blood pressure, *DISH *Depression interview and structured hamilton, *DSM-IV *Diagnostic and statistical manual of mental disorders- 4th version, *FM *Fibromyalgia, *GAD-7 *Generalized anxiety disorder 7 questionnaire, German version: ≥10 moderate to severe anxious symptomatology, *HADS *Hospital anxiety and depression scale, *LC-APCI-MS *Isotope-dilution, atmospheric pressure, chemical ionization liquid chromatography/mass spectrometry, *LC-MS/MS, *Liquid chromatography tandem mass spectrometry, *MDE *Major depressive episode, *MINI *Mini international neuropsychiatric interview, *NSAIDS *Non-steroidal anti-inflammatory drugs, *PHQ-9 *Patient health questionnaire, German version: ≥15 moderate to severe depression, *POMS *Profile of mood states

### Measurement of depression

The included studies used varying methods to diagnose depression including structured clinical interviews or self-report questionnaires. The most common measure, used in three of the included studies, was a physician-led interview using the DSM-IV criteria for MDD or the Hospital Anxiety and Depression Scale (HADS). Patient self-report scales were also used, including the 9-item patient health questionnaire (PHQ-9) [27] and 16- item quick inventory of depressive symptomatology (QIDS- SR16) [26].

Gao et al. [27] included participants in study group if they had a PHQ-9 score of > 15 (moderate to severe depression) and those with < 15 (no or mild depression) were placed in the comparison group. Stensson et al. [32] used the HADS to determine level of probable depression. They classed those who scored < 7 as a non-case, 8–10 as a doubtful case and those who scored over 11 as definite case. HADS scores were significantly different between both groups, however scores in both groups were below the > 11 cut off for probably depression (7.3±3.6 vs. 1.8±2.4). Similarly, in 2020, Stensson et al. [31] used the same HADS scale and found at baseline that participants in the study group had significantly higher HADS scores when compared to the comparison group (7.7±4.2 vs. 3.1±3.0, *p*= 0.007). Three studies used the DSM-IV to determine presence of current depressive episode and matched healthy controls with no lifetime history of psychiatric illness to these participants for the comparison group [28–30]. Finally, Barry et al. [26] found that participants with BMS had a total QIDS-SR16 score of 7.9 ± 1.3 indicating mild depression, which was also significantly higher when compared to those in the comparison group (2.5 ± 0.8).

### Assessment of endocannabinoid & N-acylethanolamine levels

Studies used varying methods to assess eCB and NAE levels. Three of the studies utilised Liquid Chromatography Tandem Mass Spectrometry (LC-MS/MS) [26, 31, 32] and a further three studies used Isotope-dilution, atmospheric pressure, chemical ionization Liquid Chromatography Mass Spectrometry (LC-APCI-MS) [28–30]. Only one study [27] collected hair samples and therefore used online solid phase extraction- Liquid Chromatography Mass Spectrometry (SPE-LC-MS) to assess the samples.

### Study findings

#### Relationship between eCB levels and incidence of depression

Levels of 2-AG in females with depression was assessed in six studies (Table 8). Three studies found 2-AG was significantly reduced [28, 29, 32], while the remaining four studies showed no significant difference in 2-AG levels compared with healthy controls [26, 27, 30, 31]. One study involving 55 females experiencing depression found an inverse relationship between 2-AG levels and duration of depressive episode (*p*= 0.05) [28].

**Table 8:** Summary of eCB results in female-sexed individuals with depression and mood disorders

First Author	Year	Study parameters	Main findings- Study population	Main findings- Comparison Group	Main findings- Study Population vs. Comparison Group [*p*-value]	Results Summary eCB levels
Barry[26]	2018	AEA, 2-AG, PEA, OEA Serum	**AEA** 0.39±0.04ng/ml **2-AG** 1.99±47ng/mol **PEA** 3.86ng/ml± 0.22 **OEA** 2.79±0.29ng/ml	**AEA** 0.32±0.03ng/ml **2-AG** 2.10±0.27ng/ml **PEA** 3.16ng/ml± 0.19 **OEA** 2.8±0.29ng/ml	**AEA***p* = 0.25 **2-AG***p* = 0.84 **PEA***p* < 0.05 **OEA***p* = 0.98	Significant positive correlation between depressive symptomatology in BMS and plasma PEA, OEA and AEA (*p* < 0.05) BMS was associated with significant increase in PEA compared to comparison group (*p* < 0.05) No significance in OEA, AEA and 2-AG between groups
Gao[27]	2020	AEA. 2-AG, PEA, OEA, SEA Hair Samples	High Symptom **AEA** 0.73 ± 0.57 **2-AG** 1.66 ± 0.18 **PEA** 3.17 ± 0.31 **OEA** 3.05 ± 0.37 **SEA** 2.90 ± 0.28	Low Symptom **AEA** − 0.59 ± 0.37 **2-AG** 1.70 ± 0.16 **PEA** 3.10 ± 0.27 **OEA** 2.97 ± 0.34 **SEA** 2.79 ± 0.24	*PHQ-9* **AEA** 0.328 **2-AG** 0.310 **PEA** 0.250 **OEA** 0.301 **SEA** 0.031	Elevated levels of SEA present in study population (*p* = 0.031) when compared to comparison group
Hill[28]	2008	AEA, 2-AG Serum	*MINOR DEPRESSION* **AEA** 0.95 ± 0.44pmol/mL **2-AG** 26.4 ± 18.32 *MAJOR DEPRESSION* **AEA** 0.74 ± 0.29 pmol/mL **2-AG** 12.5 ± 5.6pmol/mL	*MINOR DEPRESSION CONTROL* **AEA** 0.60 ± 0.18pmol/mL **2-AG** 18.18 ± 12.37 *MAJOR DEPRESSION CONTROL* **AEA** 0.72 ± 0.29 pmol/mL **2-AG** 19.6 ± 12.5pmol/mL	*MINOR DEPRESSION* **AEA***p* = 0.02 **2-AG***p* = 0.22 *MAJOR DEPRESSION* **AEA***p* = 0.84 **2-AG***p* = 0.04	***Major Depression*** 2-AG was significantly reduced in those with major depression (*p* = 0.04) compared to matched controls. However, this was not significantly correlated with total Hamilton Score A significant negative correlation between 2-AG and duration of current depressive episode (*p* = 0.05). No correlation found between those with recurrent major depression or those with their first episode (*p* = 0.90) No difference in AEA levels between those with major depression and their matched controls (*p* = 0.84) **Minor Depression** AEA was significantly increased in those with minor depression (*p* = 0.02) compared to matched controls 2-AG was higher in those with minor depression compared to controls, however this was non-significant (*p* = 0.22)
Hill[29]	2009	AEA, 2-AG, PEA, OEA Serum	**AEA** [t [27] = 2.06)] **2-AG** [t [27] = 2.40] **PEA** [t [27] = 0.10] **OEA** [t [27] = 0.67]	Unable to retrieve values from Author	**AEA***p* < 0.05 **2-AG***p* < 0.03 **PEA** > 0.05 **OEA***p* > 0.05	Under basal conditions serum AEA and 2-AG were significantly reduced in study population relative to comparison group (*p* < 0.05). No difference in PEA or OEA was present between groups
Ho[30]	2012	AEA, 2-AG Serum	**AEA** 0.8±0.1 pmol/ml **2-AG** 18.5±2.7 pmol/ml	**AEA** 0.7±0.1 pmol/ml **2-AG** 19.0±2.4 pmol/ml	**AEA***p* > 0.05 **2-AG***p* > 0.05	No difference in average serum AEA & 2-AG between groups
Stensson[32]	2018	2-AG, PEA, OEA, SEA Serum	**AEA** 0.31±0.15nM **2-AG** 14.5 ±9.2 nM **PEA** 9.5 ±2.4 nM **OEA** 6.2 ±2.3 nM **SEA** 2.6 ±1.0 nM	**AEA** 0.31±0.15nM **2-AG** 11.1±5.1 nM **PEA** 8.6±2.5 nM **OEA** 5.4±1.8 nM **SEA** 2.1±0.9 nM	**AEA***p* = 0.99 **2-AG***p* = 0.001 **PEA***p* = 0.010 **OEA***p* = 0.006 **SEA***p* = 0.001	Significantly higher levels of 2-AG (*p* = 0.001), PEA (*p* = 0.010), OEA (*p* = 0.006) and SEA (*p* = 0.001) in those with FM compared to comparison group No difference in AEA levels between those with depression and those without. Significant correlation between AEA and HAD-D in FM (*p* = 0.05) No significant correlation between 2-AG, HADS in FM
Stensson[31]	2020	AEA, 2-AG, PEA, OEA, SEA Serum	**AEA**0.26±0.13 **2-AG**12.8±8.49 **PEA**8.51±2.03 **OEA**5.66±1.92 **SEA**2.56±1.18	**AEA** 0.31±0.16 **2-AG**12.5±3.97 **PEA**8.72±1.63 **OEA**5.39±1.29 **SEA**2.05±0.83	**AEA***p* = 0.12 **2-AG***p* = 0.83 **PEA***p* = 0.63 **OEA***p* = 0.50 **SEA***p* = 0.04	SEA levels were significantly elevated in study population compared to comparison group (*p* = 0.04)

KEY* AEA* Anandamide, *BMS *Burning mouth syndrome, *BMI *Body mass index, *HAD-D* Hospital anxiety and depression scale- depression, *FM *Fibromyalgia, *OEA *Oleoylethanolamide, *PEA* Palmitoylethanolamide, *SEA *N-stearoylethanolamine, *2-AG* 2-arachidonoylglycerol, *2-OG* 2-oleoylglycerol

Similarly inconsistent results were reported with regards to AEA levels (Table 7); levels of AEA significantly increased in females with minor depression compared to matched controls in one study (0.95 ± 0.44 vs. 0.60 ± 0.18, *p* = 0.02) [28], while one study found AEA was significantly reduced in depressed females ([t [27] = 2.06)], *p*< 0.05) [29]. Other studies found no significant difference in AEA levels between females with depression compared to healthy controls [26, 30, 31].

Three studies found significantly higher levels of SEA in depressed females (2.90 ± 0.28 vs. 2.79 ± 0.24, *p* = 0.031 [27], 2.61 ± 1.00 vs. 2.1 ± 0.9, *p* = 0.01 [32], 2.56±1.18 vs. 2.05±0.83, *p* = 0.04) [31] compared to healthy controls. Both studies which investigated PEA found significantly higher levels of PEA in females diagnosed with depression (3.86±0.22, *p* < 0.05^26,^ 9.5 ±2.4 vs. 8.6±2.5, *p* = 0.010).^32^ A significantly higher level of OEA was also present in depressed females with FM (6.2 ±2.3 vs. 5.4±1.8, *p*= 0.006) [32].

#### Correlation between eCB levels and depression severity

Two studies reported a significant positive correlation between AEA, PEA and OEA levels and depression symptomatology, suggesting that the severity of depressive symptoms is influenced by altered levels of eCBs and thus indicates involvement of the ECS in mood disorders [26, 32]. Stensson et al. [32] found no association between 2-AG and clinically relevant depression, while Hill et al. [28] observed no difference in 2-AG between those in their first depressive episode compared to those with recurrent major depression. However, Hill et al. [28] did find a significant negative correlation between 2-AG levels and the duration of current depressive episodes, however reduction in serum 2-AG content was not correlated with Hamilton scores suggesting that this reduction in 2-AG is not as a result of depression (Table 8).

## Discussion

MDD is a mental health condition with widely debated pathophysiology, that is still poorly understood and under-studied in the context of female individuals [7]. A defining factor that influences the development of MDD is a dysregulation of the ECS and its endogenous ligands, eCBs and NAEs [10]. The evidence identified through this systematic review suggest altered levels of eCBs and NAEs are present in depressed females. However, no cohesive pattern was identified, with studies observing both reduced and elevated levels of eCBs and NAEs. The variability in study designs from screening tools used for depression, eligibility criteria and analysis methods of eCBs unfortunately lacked the consistency needed to attain a reliable and valid understanding of eCB levels in females with depression.

The included studies used a wide range of assessment tools to confirm and quantify depression, including both self-report screening measures and structured clinical interviews. Difficulties arise when comparing results across studies that use different depression screening measures, as each have a unique set of questions and cut-off bands to quantify depression. Concerns have also been raised about the difference in cut off bands used by self-report questionnaires to measure severity of depressive symptoms. When comparing use of HADS and PHQ-9 in a clinical setting, 74% of individuals assessed by PHQ-9 would be offered an anti-depressant, while only 37% would be written a prescription when assessed by HADS [33]. Self-reported measures were also used to sort participants into groups, with levels of eCBs being compared amongst groups with minor or “less severe” depression [27, 28]. Mild depression is only a less severe variant of major depression, and comparing groups with the same illness may be limit the ability to accurately determine the difference in eCB levels between groups. In contrast, structured clinical interviews using the DSM-IV criteria is currently considered the gold standard for assessing depression [34] and is the suggested tool that researchers should use to assess their study populations [35]. However, it should also be recognised the influence that sex and gender plays in MDD, with concern’s raised about the DSM-V classification of MDD, particularly as the criteria does not distinguish between men and women. Experience of MDD between sexes has been established, with females more likely to report anxiety, somatic symptoms and guilt compared to males. Lack of inclusion from female-sex perspectives and social determinants in the DSM-V classification of MDD, raises the question if diagnostic criteria should be further evaluated to incorporate sex-based differences to improve identification and treatment of MDD. Future studies with the objective to compare levels between healthy populations and those with a mental health conditions should also aim to ensure appropriate screening of participants with standardised diagnostic procedures and allocate a true healthy population as the comparator.

Enrolment of participants with other co-morbid diseases, namely FM and BMS presented another methodological confounder. Literature has found altered levels of eCB in both of these conditions; eCB deficiency has been proposed as a cause of FM due to its role in pain processing and inflammation [36, 37]. Individuals with BMS have altered TRPV1 and CB1 receptors expression on tongue epithelia, suggesting that the ECS plays a role in the pain and severity of symptoms in BMS [38]. Enrolling participants with diagnoses that have underlying pathophysiology linked to ECS dysfunction hinders the ability to determine if variations in eCB levels are due to depression or to another disease. Furthermore, Ho et al. [30] enrolled participants with concurrent depression and heart disease, finding that AEA and 2-AG levels in females with depression was positively correlated with diastolic and mean arterial pressure. The authors suggested that the biochemical changes in depression, such as increased blood pressure, lends itself towards eCBs playing a key faciliatory role in both diseases to reduce the extent of hypertension and it’s associated inflammation [30]. Again, this study was not able to determine if the altered eCB levels was a result of heart disease or presence of depression. Thus, it is essential that future research aimed at assessing the role of the eCB in depressive disorders excludes participants with concurrent diseases in which the ECS is already implicated. To gain further understanding of the ECS in other conditions, a review may be warranted to identify these to understand the mechanisms of the ECS and the potential variations in eCB levels.

**Fig. 2:**
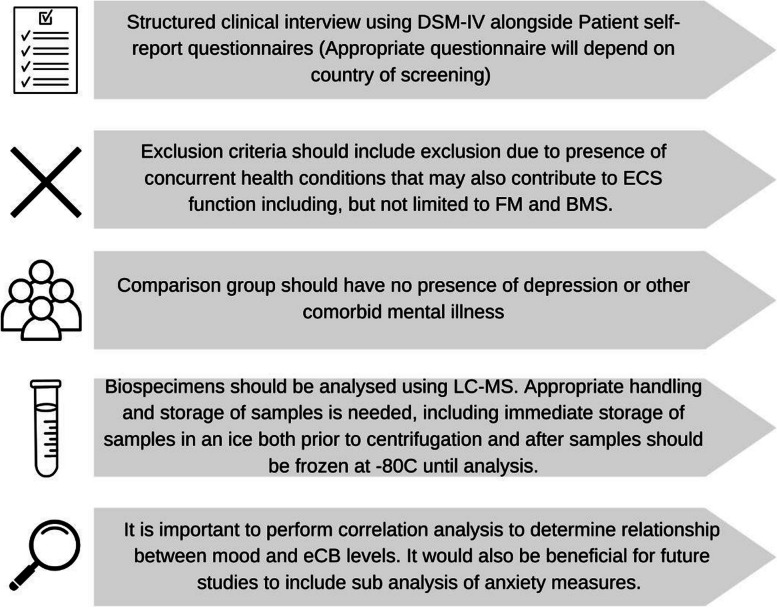
Suggested methodological changes for future studies looking to asses eCB and NAE levels in females with mood disorders

Methods of measuring eCBs and storing samples also varied between studies. LC-MS is most commonly used to determine levels of eCBs in blood samples due to its high sensitivity, high validity and short sample preparation time [39]. While variations of LC-MS, including using atmospheric pressure chemical ionization or electrospray ionisation, are routinely used for measuring compounds found in bodily fluids, there is no known research comparing the efficacy and validity of these testing methods in context of eCBs. It is essential that appropriate storage of collected blood samples are used for eCBs measurements [39]. For clinical studies, it is highly recommended that anti-coagulated blood is immediately placed in an ice bath, and then centrifuged before being frozen and stored at -80^o^C until analysis [40]. eCBs are highly sensitive to temperature variation; it has been found that whole blood storage at 4^o^C can increase AEA concentrations in the sample [40]. Only one study in this review reported storing blood samples at -80^o^C [30], with four storing samples at -70^o^C [28, 29, 31, 32] and one not disclosing any blood sample handling or storage procedures [26]. When collating results that assess eCB levels in blood samples, researchers should critically analyse blood sample collection and handling techniques in order to draw appropriate conclusions and limit confounding factors from data provided. It is also essential that future studies that assess eCB levels use standardised procedures of blood sampling and to ensure upmost accuracy of eCB measurement and data reporting [40]. There is less data on the validity of measurement of eCBs in hair samples, however hair samples have been suggested to be a highly sensitive indicator of retrospective quantification of eCB levels in those with depression, further research is still needed to fully attain their reliability [27]. Refer to Fig. 2 for an outlines of suggestions to improve methodology in future studies.

The ECS has a documented role in regulating homeostasis of physiological, cognitive and emotional processes in the body, suggesting a possible link between ECS dysfunction and the development of mood disorders in females [41]. Proposed aetiologies of MDD such as genetic influences and inflammation, are both intrinsically linked with the ECS. Firstly, a genetic study of predominantly female participants concluded that CB1 receptor haplotypes increased the individual’s risk of depression after experiencing adverse life events, which may explain the higher ratio of females who are diagnosed with depression [42]. Secondly, increased levels of inflammatory cytokines IL-8, IFN-y, TNF-α and CRP have been positively correlated to depressive symptoms in females [43, 44]. Interestingly, 2-AG and AEA have shown anti-inflammatory activity by down regulating inflammatory cytokines such as TNF-α, suggesting that the ECS may play a role in modulating neuroinflammation associated with depression [18]. Finally, preclinical reports have suggested that modulation of the ECS displays similar actions to pharmaceutical anti-depressants by enhancing serotonergic and noradrenergic transmission resulting in anti-depressant effects [23].

Although beyond the scope of this review, the included studies did highlight the potential influence of stress and anxiety on eCB levels, both conditions which are closely interlinked with the severity of depression in females [28]. It is important not to overlook that mental health disorders often accompany each other and it is essential to understand the relationships between depression and anxiety/stress in order to provide appropriate therapeutic treatment. Approximately 41.6% of individuals with MDD have been found to have a concurrent anxiety disorder over the same 12-month period [45]. The presence of significant anxiety with MDD has often been found to be a predictor of more severe depression and increased suicidal ideation [45]. The involvement of AEA in anxiety has been shown in both human and animal studies in which a significant reduction in anxiety was correlated with increased AEA in female-sexed individuals with clinically relevant anxiety. Interestingly, animal studies have shown that AEA metabolism elicits anxiolytic effects, perhaps providing a novel target for anxiolytic in human individuals [28]. Further exploration into the relationship between anxiety & depression in females and the ECS is warranted, which could provide insight into screening and treatment for females with comorbid anxiety and depression. Although out of scope for this review, future research examining the function of the ECS in females with other psychiatric disorders like bipolar or schizophrenia would be of interest.

### Limitations

There are several limitations to this research that need to be considered. As an emerging area of research our search criteria may not have identified all available literature thus limiting the amount of included studies. We excluded one study due to an inability to translate [46]. Due to the limited amount of data available, pooled analysis of data was not possible and each study was critically analysed as a standalone study.

## Conclusion

This systematic review offers a spectrum of results and insights into the potential role of the ECS in females with MDD. Despite the varied outcomes, there appears to be a trend supporting a hypoactive ECS is implicated in females with MDD. However, it still remains unclear whether altered levels of eCBs are causal, co-occurring or as a consequence of depression. The limited number of studies included in this review exacerbates the conflicting results and underscore the lack of research specifically focusing on the ECS, females and MDD. Furthermore, lack of available data focusing on the role of the ECS in females warrants future research to gain a deeper understanding of pathophysiological mechanisms. Future studies should prioritise the use of validated self-report measures and structured clinical interviews to diagnose depression, establish appropriate inclusion criteria’s, standardise blood handling and storage methods and conduct sub-analysis of stress and anxiety measures to comprehensively elucidate the role of the ECS in females with MDD.

## Data Availability

The data and material used during this systematic review are available upon request. Please contact Meagan McWhirter by emailing m.mcwhirter.11@student.scu.edu.au.
